# LTR retrotransposons in rice (*Oryza sativa*, L.): recent burst amplifications followed by rapid DNA loss

**DOI:** 10.1186/1471-2164-8-218

**Published:** 2007-07-06

**Authors:** Clémentine Vitte, Olivier Panaud, Hadi Quesneville

**Affiliations:** 1 Laboratoire Ecologie, Systématique et Evolution, Université Paris Sud, Orsay, France; 2 Laboratoire Bioinformatique et Génomique, Institut Jacques Monod, Paris, France; 3 Laboratoire Génétique et Développement des Plantes, Université de Perpignan, Perpignan, France; 4 Bennetzen laboratory, University of Georgia, Athens, GA, USA

## Abstract

**Background:**

LTR retrotransposons are one of the main causes for plant genome size and structure evolution, along with polyploidy. The characterization of their amplification and subsequent elimination of the genomes is therefore a major goal in plant evolutionary genomics. To address the extent and timing of these forces, we performed a detailed analysis of 41 LTR retrotransposon families in rice.

**Results:**

Using a new method to estimate the insertion date of both truncated and complete copies, we estimated these two forces more accurately than previous studies based on other methods. We show that LTR retrotransposons have undergone bursts of amplification within the past 5 My. These bursts vary both in date and copy number among families, revealing that each family has a particular amplification history. The number of solo LTR varies among families and seems to correlate with LTR size, suggesting that solo LTR formation is a family-dependent process. The deletion rate estimate leads to the prediction that the half-life of LTR retrotransposon sequences evolving neutrally is about 19 My in rice, suggesting that other processes than the formation of small deletions are prevalent in rice DNA removal.

**Conclusion:**

Our work provides insights into the dynamics of LTR retrotransposons in the rice genome. We show that transposable element families have distinct amplification patterns, and that the turn-over of LTR retrotransposons sequences is rapid in the rice genome.

## Background

Transposable elements (TEs) make up a large part of eukaryotic genomes. They represent a genomic fraction of 3% in baker's yeast [1], ~20% in fruit fly [2-5], 45% in human [6,7] and over 80% in maize [8,9]. Due to their repetitive nature and to the fact that they harbor regulatory signals, TEs are responsible for chromosomal rearrangements [10], fragmental gene movements [11,12] and for the evolution of gene regulation and function [13,14]. Hence, the activity of TEs is currently considered to be one of the major processes in genome evolution.

In plants, Long Terminal Repeat (LTR) retrotransposons are the most common type of TE: they are ubiquitous in the plant kingdom [15] and are the main constituents of large plant genomes [15,16]. Moreover, these elements have been shown to be responsible for wide genome expansions [8,9,17-21] and are considered to be major players in the remarkable variation of genome size observed in flowering plants [22,23], along with polyploidy.

LTR retrotransposons are class I TEs and thus replicate using a RNA intermediate, through a "copy-and-paste" mechanism. They are related to retroviruses with which they share their structure: the complete copies consist of two LTRs that flank an internal region. LTR sequences contain the signals for transcription initiation and termination, while the internal region encodes the proteins that are necessary for the retrotransposition cycle. LTR retrotransposons are classified into two major families: the *Ty1*/*copia*-like and *Ty3*/*gypsy*-like elements.

Plant LTR retrotransposons vary in size from 2 to 18 kb and harbor LTRs that vary in size from a few hundreds bases to several kilobases [15,24]. LTRs are terminated by a short inverted dinucleotide, usually 5'-TG-3' and 5'-CA-3' [15]. Their well-defined structure, their role in genome size expansion, their prevalence and their repetitive nature make LTR retrotransposons good models to study genome evolution. In all plants analyzed, LTR retrotransposons appear to have undergone recent amplifications (*i.e. *within the past 15 million years) [9,25-34]. LTR retrotransposons are nevertheless present in all plant lineages and thus of ancient origin (reviewed in [15] and [24]).

The distribution and structure of LTR retrotransposons have been studied in several species, in particular in the two model plants for which a nearly complete genomic sequence is available: *Arabidopsis thaliana *[25-27,30,31] and rice (*Oryza sativa *L.) [28,32,33]. In rice, however, these studies have been based on either numerous LTR retrotransposon families but within a relatively small portion of the genome [28,32] or small subsets of LTR retrotransposon families within approximately the entire genome [33]. In our study, a set of 41 LTR retrotransposon families was analyzed on the almost complete rice genome (~365 Mb). This comprehensive structural analysis provides insights into both the amplification and the subsequent elimination of LTR retrotransposon sequences and reveals the highly dynamic nature of the rice genome.

## Results and discussion

### Global analysis of 41 LTR retrotransposon families: copy number, current genomic fraction and DNA amount deleted since their insertion

We extracted the paralogous copies of 41 LTR retrotransposons families (16 *gypsy*-like and 25 *copia*-like families, Table 1) from the rice genome sequence using Blaster, a program suite based on the Blast program [35] and improved for the detection of transposable elements [4]. Through this initial Blaster search, we retrieved from the rice genome sequence more than 13,000 LTR retrotransposon copies, with copy numbers per family ranging from a few copies to over 2000 copies per haploid genome (Table 2). Because the total sequence of the 12 rice pseudomolecules analyzed represents only ~94% (365 Mb) of the rice genome, we believe, however, that the copy numbers of these families could possibly be higher.

**Table 1:** Description of the 41 reference copies and detection of the LTR position

**Family name**	**Class**	**Total size**	**LTR size**
***Dagul***	*Gypsy*	13425	3623
***Hopi/Osr27***	*Gypsy*	12892	1103
***Houba/Tos5/Osr13***	*Copia*	6437	968
***Osr1***	*Copia*	6400	965
***Osr2***	*Copia*	4950	268
***Osr3***	*Copia*	5201	146
***Osr4***	*Copia*	5665	350
***Osr5***	*Copia*	6092	477
***Osr6***	*Copia*	5204	440
***Osr7***	*Copia*	8908	1618
***Osr8***	*Copia*	9222	1220
*Osr9*	*Copia*	2766	RT only (28)
*Osr10*	*Copia*	1821	RT only (28)
*Osr11*	*Copia*	1114	RT only (28)
***Osr12***	*Copia*	4719	221
***Osr14***	*Copia*	8371	303
***Osr15***	*Copia*	5063	255
*Osr16*	*Copia*	6908	ambiguous LTR position
***Osr17***	*Copia*	5957	466
*Osr18*	*Copia*	1614	ambiguous LTR position
***Osr19***	*Copia*	4707	172
***Osr20***	*Copia*	5509	286
***Osr22***	*Copia*	4737	159
*Osr23*	*Copia*	4474	ambiguous LTR position
***Osr24***	*Copia*	4852	221
***Osr28***	*Gypsy*	18007	2195
***Osr29***	*Gypsy*	9007	656
***Osr30***	*Gypsy*	13001	1507
*Osr31*	*Gypsy*	7403	ambiguous LTR position
***Osr34***	*Gypsy*	12796	3292
***Osr35***	*Gypsy*	5688	423
***Osr36***	*Gypsy*	5157	319
***Osr37***	*Gypsy*	4436	794
***Osr38***	*Gypsy*	5511	312
***Osr39***	*Gypsy*	5217	368
***Osr40***	*Gypsy*	11421	564
***Osr42***	*Gypsy*	5605	358
***Osr43***	*Gypsy*	1794	291
***Osr44***	*Gypsy*	1200	148
***RIRE1***	*Copia*	8322	1523
***Tos17/Osr21***	*Copia*	4204	138

The elements highlighted in bold correspond to the elements for which the LTR position was validated after comparison of the literature data with our annotation. Total size of the element is given after homogenization of the two LTRs (see Materials and Methods). Data extracted from the literature are from [28], except for *RIRE1 *[44] and *Tos17/Osr1 *[45].

**Table 2:** Global analysis of the Blaster output

		**Results from local alignment/reference copy**											
**Family name**	**Copy nb.**	**Element size (% of ref. copy size)**						**Seq. identity/ref. copy**					
		**Min.**	**1^st ^Q.**	**Med.**	**Mean**	**3^rd ^Q.**	**Max.**	**Min.**	**1^st ^Q.**	**Med.**	**Mean**	**3^rd ^Q.**	**Max.**
***Osr34***	2107	0.2	1.0	3.8	13.2	12.6	99.3	0.65	0.82	0.87	0.87	0.91	1.00
***Osr30***	1757	0.2	1.1	4.6	15.6	11.4	100.0	0.64	0.83	0.86	0.86	0.88	1.00
***Hopi/Osr27***	1332	0.2	0.5	8.1	29.4	54.2	99.6	0.65	0.85	0.90	0.89	0.95	1.00
***Dagul***	1052	0.2	0.7	4.2	17.1	27.2	100.0	0.66	0.87	0.90	0.90	0.94	1.00
*Osr31*	986	0.3	1.4	1.9	2.2	2.5	92.4	0.66	0.85	0.87	0.87	0.90	1.00
***Osr14***	934	0.3	2.5	10.3	16.1	27.5	100.0	0.67	0.85	0.89	0.88	0.91	1.00
***Osr40***	855	0.2	1.4	2.9	22.4	28.5	100.0	0.65	0.82	0.86	0.85	0.90	1.00
***Osr8***	831	0.4	3.6	11.0	21.3	19.9	99.9	0.65	0.85	0.89	0.88	0.92	1.00
***Osr37***	565	0.6	3.1	5.0	26.6	49.0	100.0	0.67	0.84	0.86	0.86	0.88	1.00
***Houba/Tos5/Osr13***	563	0.3	4.9	14.9	41.4	96.7	100.0	0.63	0.89	0.94	0.93	0.98	1.00
***Osr29***	446	0.3	3.3	7.1	25.7	37.6	100.0	0.65	0.84	0.87	0.87	0.91	1.00
***Osr1***	269	0.4	1.5	15.1	34.5	69.5	100.0	0.72	0.92	0.94	0.94	0.97	1.00
***RIRE1***	262	0.7	3.1	5.6	14.8	6.7	87.9	0.70	0.84	0.85	0.85	0.86	0.93
***Osr15***	188	0.5	2.0	5.1	19.3	10.8	100.0	0.68	0.87	0.90	0.90	0.94	1.00
***Osr17***	187	0.5	1.1	4.2	31.1	87.2	100.0	0.78	0.87	0.92	0.91	0.95	1.00
*Osr23*	145	0.5	2.6	4.7	11.8	9.5	100.0	0.71	0.78	0.81	0.81	0.84	1.00
*Osr9*	119	1.5	7.3	49.1	48.4	87.6	100.0	0.73	0.79	0.84	0.86	0.95	1.00
*Osr16*	111	0.6	0.7	0.8	2.9	1.5	100.0	0.71	0.89	0.92	0.91	0.94	1.00
***Osr4***	81	0.4	18.2	93.5	64.7	95.2	100.0	0.68	0.94	0.96	0.95	0.98	1.00
*Osr10*	75	1.4	33.0	98.5	71.8	100.0	100.0	0.70	0.94	0.96	0.93	0.97	1.00
***Osr19***	75	0.4	1.5	2.6	9.8	4.1	100.0	0.75	0.81	0.83	0.84	0.86	1.00
***Osr24***	73	0.6	1.1	2.7	10.1	4.5	100.0	0.73	0.80	0.82	0.83	0.86	1.00
***Osr7***	69	0.4	0.7	1.5	20.4	17.6	100.0	0.70	0.86	0.93	0.91	0.95	1.00
***Osr22***	68	0.5	1.5	2.8	11.3	3.8	87.9	0.73	0.79	0.82	0.82	0.85	1.00
***Osr28***	58	0.2	0.5	1.4	10.1	5.8	100.0	0.63	0.86	0.89	0.88	0.92	1.00
***Osr39***	57	1.0	3.1	4.9	11.9	13.1	100.0	0.69	0.79	0.81	0.81	0.82	1.00
***Osr5***	52	0.9	3.2	11.8	22.2	24.7	100.0	0.73	0.81	0.89	0.88	0.96	1.00
***Osr3***	51	1.3	3.4	19.9	32.9	52.5	100.0	0.65	0.79	0.82	0.82	0.85	1.00
***Osr43***	50	3.0	7.5	8.1	20.6	16.8	100.0	0.72	0.84	0.90	0.89	0.94	1.00
*Osr18*	34	2.5	5.3	12.6	26.3	37.0	100.0	0.72	0.79	0.82	0.83	0.85	1.00
***Osr36***	34	1.2	2.0	2.9	17.7	14.0	100.0	0.76	0.82	0.85	0.86	0.89	1.00
***Osr6***	27	1.6	4.5	51.5	43.5	77.0	100.0	0.73	0.77	0.82	0.83	0.86	1.00
***Tos17/Osr21***	26	1.7	2.0	5.6	24.0	38.4	100.0	0.64	0.76	0.82	0.83	0.88	1.00
*Osr11*	25	11.8	70.6	90.1	74.4	93.1	100.0	0.81	0.83	0.85	0.86	0.88	1.00
***Osr42***	21	0.8	1.5	2.4	13.6	3.7	100.0	0.74	0.80	0.82	0.83	0.86	1.00
***Osr35***	18	1.0	2.0	2.6	12.9	7.2	100.0	0.73	0.79	0.82	0.83	0.84	1.00
***Osr44***	18	3.4	8.0	10.6	22.5	21.3	99.9	0.81	0.88	0.92	0.91	0.94	1.00
***Osr12***	16	1.3	2.1	2.4	28.0	57.1	100.0	0.83	0.86	0.88	0.91	1.00	1.00
***Osr2***	13	1.0	1.5	2.8	29.4	70.1	99.3	0.84	0.90	0.92	0.92	0.94	1.00
***Osr38***	13	1.4	2.0	3.2	16.5	10.6	89.3	0.75	0.81	0.81	0.84	0.86	1.00
***Osr20***	11	1.2	2.3	5.5	29.6	52.2	99.9	0.82	0.91	0.94	0.92	0.96	1.00

The elements highlighted in bold correspond to the elements for which the LTR position was validated (see Table 1). ref.: reference; seq.: sequence; nb.:number; ins.: insertion; Min.:minimum; Q.:quartile; Med.:median; Max.:maximum

Altogether, these elements represent 7.8% (30.4 Mb) of the current rice genome. If all the copies mined correspond to real paralogous copies of the families and if their size upon insertion was similar to that of their corresponding reference copy, they represented 136.8 Mb upon insertion, suggesting that 106.4 Mb (77.8%) of the DNA conferred by their insertion has been removed.

The analysis of the mined copies revealed that some reference copies harbor insertions, most of them smaller than 200 bp, and a few larger than 200 bp. It also revealed that one family shares LTR with a LArge Retrotransposon Derivative (LARD). To correct for the possible detection of false paralogous copies, we therefore applied a filter discarding any fragment smaller than 200 bp, and did not take into account families with larger inserts or other problematic features (see Material and Methods). With this new filter, we estimate that 5154 copies from 19 families, which represented 52.3 Mb upon insertion, remain today in the rice genome as 20.2 Mb, leading to a loss of 32.1 Mb (61.4%).

We believe that the first data are an over-estimate and that the latter may be an under-estimate of the total percentage of DNA eliminated from the rice genome. Hence, we estimate that the percentage of DNA that has been eliminated from the rice genome since the insertion of the detected copies is comprised between 61% and 78%.

To get an overview of the deletion process for each family, we computed the distribution of the copy size relative to the reference copy size (Table 2). This analysis revealed that, for all families, most of the copies are highly truncated (most of the families have a median length below 15%). In addition, since each genomic copy shows a high sequence identity with its corresponding reference copy (median range: 0.81–0.96), all the copies belonging to a given family also share high sequence identity. Taken together, the high degree of deletion and the high level of identity between copies within a family suggest that LTR retrotransposon copies start being eliminated from the genome shortly after their insertion.

### Rice LTR retrotransposons amplify in burst-like patterns which differ among families

To provide a global overview of the LTR retrotransposon sequence turn-over process in the rice genome, we first wanted to estimate the insertion dates of the largest set of copies possible, for each family.

For copies with 2 LTRs, the best method to estimate insertion date is based on the divergence between the two LTRs of each copy [9]. For copies lacking one or two LTRs (*i.e*., truncated copies of LTR retrotransposons or copies of other types of TEs such as LINEs or DNA transposons), the method currently used is based on pairwise nucleotide identity between elements that are closely related at the phylogenetic level or between genomic copies and a consensus of the element [36-38]. However, this method estimates the overall insertion date of the set of copies as a whole, and cannot estimate the insertion date for each individual genomic copy. Moreover, consensus-based methods greatly depend on the quality of the consensus and thus on the selection of the copies used to build it.

**Figure 1:**
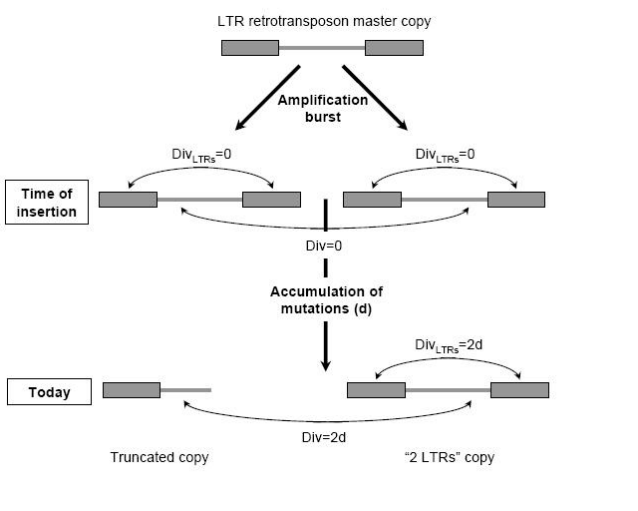
**New method to estimate the insertion date of truncated copies**. When a burst of amplification occurs, all the copies deriving from one master copy insert approximately at the same time. Upon insertion, all the new copies are identical in sequence as well as the two LTRs of each copy, leading to a null divergence between copies (Div = 0) and between the two LTRs of each copy (Div_LTRs _= 0). Over time, all sequences evolve at the same rate, so both the divergence between two copies and the divergence between the two LTRs of one copy are equal at a given time. Hence, if the nucleotide divergence between a truncated copy and a copy with two LTRs ("2 LTRs" copy) is equal to the nucleotide divergence between the two LTRs of the "2 LTRs" copy, the two copies originated from the same burst of amplification and the insertion date estimated for the "2 LTRs" copy can be used as an approximation of the insertion date of the truncated one.

To overcome these caveats, we designed a new method (see Materials and Methods and Figure 1) to estimate the insertion date of the truncated copies. As we showed in a previous work on three rice *gypsy*-like LTR retrotransposons [29], rice LTR retrotransposons amplify in bursts, leading to the insertion of many related copies in a short period of time. If this is true for all rice retrotransposons, it should be possible to find, for each truncated copy, a copy with two LTRs that originated from the same amplification burst, and has approximately the same age. The age of a given truncated copy can then be estimated using both the age of the most related copy with two LTRs, and the sequence identity existing between this complete copy and the truncated one (Figure 1).

**Figure 2:**
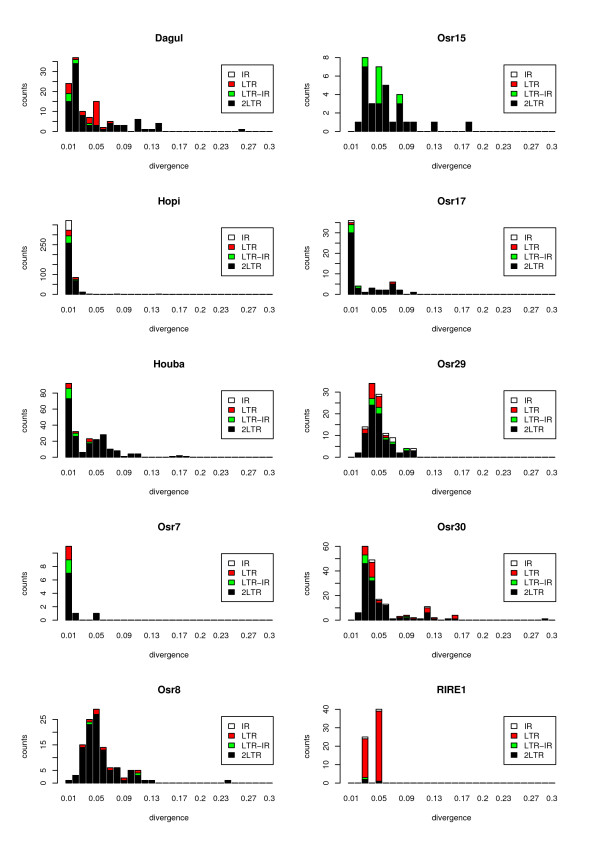
**Histogram of the copy number by date of insertion for 10 LTR retrotransposon families**. The LTR divergence is an estimate of the insertion date of the copies, each time scale corresponding to a LTR divergence of 0.01 (0.385 My). For complete copies, ("2 LTRs") this divergence corresponds to the divergence between the two LTRs. For truncated copies (Internal Region [IR], LTR, and LTR-Internal region [LTR-IR]), the divergence is derived from the "2 LTRs" copies (see Materials and Methods and Figure 1).

By applying this method on the complete dataset of all genomic copies detected (after filtering by length), we could analyze the amplification timing of 10 LTR retrotransposon families (6 *copia*-like and 4 *gypsy*-like), for which an insertion date could be estimated for more than 15 copies. The histograms of these data, presented in Figure 2, show at least one peak per family, revealing that the 10 families have all undergone at least one burst of amplification. These bursts, however, vary in extent and age between families. Some families such as *Hopi*/*Osr27*, *Osr17*, *Houba/Osr13 *and *Osr7 *have undergone very recent amplification (divergence less than 0.01, *i.e. *less than 0.4 My), whereas that of other families, such as *Osr15*, *Osr29*, *Osr30*, *Osr8*, *RIRE1 *(divergence around 0.05, corresponding to 1.9 My, or *Osr30 *(around 0.03, corresponding to 1.2 My) is more ancient. Some families, such as *Dagul *or *Houba/Osr13*, seem to have undergone several bursts of amplification, since several peaks are noticeable. The dates of these peaks vary between families from around 0.02 and 0.05 for *Dagul *to 0.01 and 0.04–0.06 for *Houba/Osr13*. Some of the older peaks, such as the second peak for *Dagul*, could only be observed through the addition of the dated truncated copies, illustrating that estimating the insertion date of truncated copies is important to describe the amplification process LTR retrotransposons in rice as accurately as possible.

In terms of magnitude, some families, such as *Hopi/Osr27*, *Osr30 *or *Houba/Osr13*, have undergone large bursts of amplification, involving over 350, 100 and 80 copies per burst, respectively. Interestingly, *Dagul*, *Hopi/Osr27 *and *Houba/Osr13*, which are among the 5 most recently amplified elements, were previously characterized by our team using the subtractive "Representational Difference Analysis" (RDA) cloning technique between rice and foxtail millet [39], and were subsequently shown to have inserted recently in the rice genome [29,39]. This suggests that the RDA technique mainly allows the extraction of young highly repeated sequences. The new data concerning these three elements confirm those that we obtained for 30% of the rice genome [29]. In particular, they confirm that the amplification burst of *Hopi/Osr27 *was large and rapid, and took place in a very recent past [29] or may still be actively transposing.

### The elimination of LTR retrotransposon sequences is efficient in rice, with a half-life of 3–4 My

As shown above, we estimate the amount of LTR retrotransposon DNA removed from the rice genome around 61–78%. Considering that most of this removal occurred within the last 5 My (most of the copies have inserted in this time lap), we estimate the half-life of a LTR retrotransposon sequence to be comprised between 3 My and 4 My. In a recent paper, Ma and collaborators [33] estimated the half-life of a retrotransposon sequence in the rice genome to be less than 6 My, using the pairwise comparison method for global insertion date estimation of the copies and a conservative substitution rate that is half as fast as ours (3.5 × 10^-9 ^substitutions per site per year versus 1.3 × 10^-8 ^in our case). After adjusting their results with the substitution rate we used, our results on a large sample are in agreement with a half-life of 3 My. In both studies large deletions could not be taken into account in the elimination process. Since such deletions could induce the elimination of one or more complete elements, we believe that the elimination of LTR retrotransposons sequences in the rice genome is even more efficient than a 3 My half-life.

To complete our analysis of the elimination process, we classified the copies according to their truncation level, into copies that harbored two LTRs, one LTR and the internal region, only one LTR, and only the internal region (designated as "2 LTRs", "LTR-IR", "LTR" and "IR", respectively). Results of this analysis, shown in Table 3, reveal that most of the copies are highly truncated. For all families except *Osr2 *and *Osr12 *(where the dataset was smallest), the copies harboring two LTRs represent less than 50% of the total number, with a median at 18.9%.

**Table 3:** Number of copies classified by truncation level (after filter)

**Family name**	**Genomic copies**								**Genomic copies with insertion date**					
	**2 LTRs**	**LTR-IR**	**IR**	**LTR**	**Total Frag.**	**Total**	**LTR/Frag.**	**2 LTRs/Total**	**2 LTRs**	**LTR-IR**	**IR**	**LTR**	**Total Frag.**	**Total**
*Osr12*	5	1	0	0	1	6	0%	83%	5	1	0	0	1	6
*Osr2*	3	2	0	0	2	5	0%	60%	1	0	0	0	0	1
*Houba/Tos5/Osr13*	206	105	14	113	232	438	49%	47%	199	20	0	15	35	234
*Osr17*	50	37	20	5	62	112	8%	45%	49	5	1	2	8	57
*Hopi/Osr27*	357	215	177	143	535	892	27%	40%	338	45	49	39	133	471
*Osr15*	28	40	4	0	44	72	0%	39%	27	6	0	0	6	33
*Osr7*	9	10	2	8	20	29	40%	31%	9	2	0	0	2	11
*Osr36*	4	1	8	0	9	13	0%	31%	4	1	0	0	1	5
*Osr29*	85	143	49	82	274	359	30%	24%	79	9	6	15	30	109
*Osr6*	4	3	12	2	17	21	12%	19%	3	0	0	0	0	3
*Osr8*	122	179	41	310	530	652	58%	19%	104	2	0	8	10	114
*Osr35*	1	2	3	0	5	6	0%	17%	1	0	0	0	0	1
*Dagul*	91	141	52	333	526	617	63%	15%	87	7	0	25	32	119
*Osr30*	143	261	93	497	851	994	58%	14%	127	11	5	32	48	175
*Osr42*	1	1	5	0	6	7	0%	14%	1	0	0	0	0	1
*Osr3*	5	15	16	0	31	36	0%	14%	5	1	0	0	1	6
*Osr28*	4	3	3	23	29	33	79%	12%	3	1	0	0	1	4
*Osr38*	0	2	4	0	6	6	0%	0%	0	0	0	0	0	0

Frag.: fragment; LTR: Long Terminal Repeat; IR: Internal Region.

Using these data along with the timing data obtained for the families (Figure 2), we found that the percentage of truncated/complete copies is correlated with the age of the family, with the younger families having fewer truncated copies than the older ones. For instance, *Hopi/Osr27*, which has amplified mainly within the last 0.4 My (LTR divergence < 0.01) shows 40% of complete copies, whereas for *Osr8*, which has amplified around 1.5–2 My ago (LTR divergence mainly within the range 0.04–0.05), these copies represent only 18.7%. These results are in agreement with previous studies, in which the same correlation was found [33]. This feature, however, cannot be viewed on Figure 2, because no insertion date could be estimated for most ancient copies.

In addition, the proportion of the different types of truncated copies over time scale increments of 0.01 (~0.4 My), presented in Figure 2, reveals very young truncated copies (e.g. for families *Dagul*, *Hopi/Osr27*, *Osr17 *and *Houba/Osr13*), revealing that the elimination of LTR retrotransposon sequences is very efficient in the rice genome.

### Processes of DNA removal: solo LTRs and accumulation of deletion analysis

The global analysis of our data showed that the elimination of rice LTR retrotransposon sequences is efficient in rice (half-life of 3–4 My). But what are the mechanisms responsible for the elimination of the copies? As shown in previous studies [27,29,33], the elimination of LTR retrotransposon sequences in plants is the result of several cellular processes such as homologous recombination (leading to the formation of solo LTRs), and the accumulation of small deletions through illegitimate recombination. In our study, we believe that the "LTR" fragments have arisen from both homologous recombination and the accumulation of small deletions or larger rearrangements, and that the two other types of truncation ("LTR-IR" and "IR") have arisen from the last two processes only. To study the formation of solo LTRs and other mechanisms, we analyzed separately the temporal dynamics of (i) the "LTR" fragments and (ii) the "LTR-IR" and "IR" fragments.

#### Solo LTR formation

Our analysis of the "LTR" fragments per 0.01 time scale increment (Figure 2) revealed that the different LTR retrotransposons harbor different amounts of "LTR" sequences. For instance, *Dagul*, *Osr29 *or *Osr30 *display a large number of "LTRs" compared to the other types of truncated sequences, whereas *Houba/Osr13 *or *Osr17 *do not show as many (Table 3). The formation of solo LTRs has been proposed to occur through unequal homologous recombination [19,27] and/or double-strand break repair [24]. In both cases, both the size of the internal region and the LTR size would impact the number of solo LTRs formed: (i) since these mechanisms are based on the physical closeness of the two LTRs, the number of solo LTRs is expected to decrease with the size of the internal region, and (ii) since they are based on the presence of homology between the two LTRs, the number of solo LTRs is expected to increase with the size of LTR. Because both internal region and LTR sizes vary through time in a given copy, these points can be made only when comparing elements that show similar insertion times, for instance *Hopi/Osr27 *vs.*Osr17 *and *Osr8 *vs. *Osr29. HopiOsr27 *has a larger internal region (10686 vs. 5491 bp), and a larger LTR (1103 vs. 466 bp) than *Osr17*. Its LTR frequency is higher (27% vs. 8%). *Osr8 *and *Osr29 *have similar internal region size, but *Osr8 *has bigger LTRs (1220 vs. 656 bp) and its LTR frequency is higher (58% vs. 30%). Therefore, LTR size seems to have an impact on solo LTR formation, but not the size of the internal region, suggesting that formation of solo LTRs is a family-dependent process. More data are however needed to confirm this pattern.

#### Accumulation of small deletions

The accumulation of small deletions has been proposed to occur through illegitimate recombination, as shown by the observation of small patches of micro-homology flanking the deleted sequences [27,33,40]. To better estimate the efficiency of this process, we analyzed the deletion rate and DNA loss rate. Since illegitimate recombination is supposed to be a general mechanism in the cell, it is expected to affect similarly all types of LTR retrotransposons and truncated sequences. Therefore, under the assumption that the accumulation of deletion is similar in all families, and occurs continuously through time, we estimated the deletion rate and the deletion loss rate by combining the data obtained for 34 families using a method derived from one classically used in animals ([41,42] and Materials and Methods).

**Figure 3:**
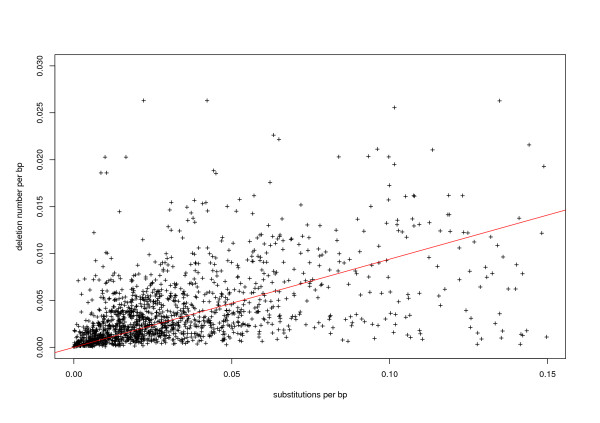
**Relationship between divergence and deletion number per nucleotide**. The slope corresponding to the maximum likelihood estimate of the deletion rate relative to substitutions (0.094) is represented in red.

Figure 3 shows the relationship between the number of deletions per bp and the number of substitution per bp. The ratio of deletion to point mutation (represented by the slope) was estimated using maximum likelihood (see Materials and Methods). Through this analysis, werevealed a deletion rate of 0.095 deletion per substitution (95% confidence limits: 0.094–0.096). With a mean deletion size of 29.28 bp (95% confidence limits: 27.15–31.69), the corresponding DNA loss rate (deletion rate times the mean deletion size) is 2.79 bp per substitution. Using this coefficient, we estimate that half of a LTR retrotransposon sequence is removed through accumulation of deletions within approximately 19 My (or 0.25 substitutions) in the absence of selection. This estimation can be compared to the DNA loss rate estimated for *D. melanogaster *[43]. In *D. melanogaster*, both mean deletion size (60.7 bp) and deletion rate (0.114) are greater, resulting in a greater DNA loss estimate of 6.9 bp per substitution. These results suggest that the removal of transposable element sequences through neutral accumulation of small deletions is more efficient in *D*. *melanogaster *than in rice, probably partially accounting for the genome size difference between these two species.

This half-life of 19 My is very different from the one we calculated based on the global size of the copies because it does not take into account selection against TE insertions and has to be considered as a quasi-neutral deletion rate estimate. Homologous recombination and selection will accelerate the elimination of TEs and thus increase the global DNA loss observed for these sequences. Moreover, with this method, we detect only the accumulation of small deletions (smaller than the copy size), and do not take into account larger rearrangements (hence, the small mean deletion size, compared to the high degree of truncation for most copies). In particular, because it is based on the analysis of the sequence pairs in the terminal tree forks, it takes into account only the newer deletions. If small deletions appear faster than large rearrangements in the rice genome, some large rearrangements may not have been taken into account by this method.

These results reveal that, even though the neutral accumulation of small deletions plays a role in LTR retrotransposons DNA removal in rice, this force is not the predominant force shaping genome size.

### Power and limits of our analyis

Any comprehensive analysis of the structure and evolution of LTR retrotransposons in a given species requires a large LTR retrotransposon database and a large genomic sequence as starting point. For our search to be as representative as possible of the whole genome, we analyzed 41 LTR retrotransposons families, including 16 *gypsy*-like and 25 *copia*-like families. As revealed by our study, these families are low, – middle- and high-copy number families (Table 1). We thus believe that this sample is representative of LTR retrotransposons in rice. The use of such a large database, coupled with the analysis of a large amount of rice sequence (~365 Mb, *i.e. *93.6% of the entire genome) allows a particularly extensive characterization of the evolutionary dynamics of LTR retrotransposons in rice. To our knowledge, this is the first time that such an extensive study is made.

The second key point is the quality of the detection. In particular, estimating how fast copies are eliminated from a genome requires to accurately mine complete copies (which can be fragmented by other insertions), as well as small fragments that have been highly truncated. The use of Blaster, a program suite based on the BLAST program [35] and improved for the detection of transposable elements [4], is particularly powerful to mine such copies. Along with the use of Blaster, the use of a database containing sequences of both the triggered LTR retrotransposons and other rice repetitive elements (see Material and Methods), allows a powerful mining of copies fragmented by other insertions. Moreover, the detection of very fragmented copies was enhanced by using the LTR retrotransposon database as subject and the genome as query in the Blaster search.

Because of these features, we believe that our method detects accurately both complete copies and truncated fragments. Since an accurate detection and estimate of the copy size is a critical point in any study on LTR retrotransposons evolutionary dynamics, we analyzed the distribution of copy size for each family.

First, we expected that the longest copy detected and the reference copy would have the same size because insertions in genomic copies (as compared to the reference copy) were not taken into account in the computation of the copy size. Results of the Blaster output, presented in Table 2, reveal that this is true for most of the families (31/41). For 6 families (*Hopi/Osr27*, *Osr2*, *Osr8*, *Osr20*, *Osr34 *and *Osr44*), the slight difference in size between the longest copy and the reference copy (99.3 to 99.9% of the reference copy size) is due to the homogenization of the two LTRs in the reference copy (see Material and Methods). For four other families (*Osr22*, *Osr31*, *Osr38 *and *RIRE1*), however, the longest copy detected is more than 5% shorter than the reference copy, which suggests that this copy was not detected by our search. *Osr22*, *Osr31 *and *Osr38 *were originally characterized by searching part of the rice genome sequence [28]. Since this sample does not completely overlap ours, it is likely that these reference copies were not included in our genomic sample. For *RIRE1*, however, the reference copy could not be detected because this sequence is not derived from a genomic copy, but is a chimeric sequence that originated from the cloning of several genomic copies [44].

Second, we expected that some short copies could be false paralogous copies of our targeted families. Mainly, we suspected that some of them could correspond to (i) uncharacterized repeated sequences inserted in the reference copy, and (ii) fragments corresponding to coding regions of uncharacterized elements sharing a high sequence identity with the element analyzed (*in silico *cross-hybridization between two different families). To check for these possibilities, we looked at the position of the mined copies on the reference copy, using the alignment that we had generated. This analysis revealed that some reference copies harbor insertions, most of them smaller than 200 bp, and a few larger than 200 bp. It also revealed that one family shares LTR with a LARD element.

Thus, we believe that such a verification step is essential to interpret the data. For instance, one may not want to perform a detailed analysis of a LTR retrotransposon that shares LTRs with a LARD element, because this feature would artefactually increase the number of solitary LTRs detected. In order not to take into account possible false paralogous copies, we applied a filter discarding any fragment smaller than 200 bp, and did not analyze the deletion process in families with larger inserts or other problematic features (see Material and Methods). This conservative strategy had mainly two draw-backs: first, some real paralogous copies with a size below 200 bp would be missed due to the filter, leading to an under-estimate of copy number and total fraction of the genome occupied by these elements. However, since the raw data most probably over-estimate these numbers, we believe that the comparison of the two outputs (with and without filter) give a good estimate of these parameters. Second, it decreased the number of families that could be used to perform a detailed analysis of the deletion process. However, by starting with a large number of 41 families, we could estimate the deletion process for 19 families, a large enough number to get an estimate that is representative of the genome.

Finally, the use of a new method to date truncated copies allowed us to get a more detailed appreciation of the integration and elimination processes than with complete copies alone, and, therefore, a more accurate estimate of temporal parameters of the deletion processes. We showed in earlier studies (29) that three rice gypsy-like LTR retrotransposons amplify through burst of amplification. Here, the use of complete copies harboring 2 LTRs revealed recent amplification bursts for several other families, suggesting that this is a general feature of rice LTR retrotransposons. Therefore, we developed a method to date LTR retrotransposon fragments based on sequence identity between truncated fragments and a complete copy that comes for the same amplification burst. This method requires the conservation of at least one complete copy per burst. Therefore, we did not expect to estimate the insertion date of all truncated copies, particularly for ancient ones, because copies of ancient burst were expected to be all truncated. It is thus possible that we missed some ancient bursts. However, this should not have any impact on the detection of the more recent bursts that we detect.

Because old copies are more likely to be deleted than new ones, this technique revealed ancient amplification events, thus improving our characterization of the amplification process. Moreover, the estimation of the insertion date for all types of copies allowed us to analyze the number of truncated copies per time period, leading to a more accurate characterization of the deletion process. However, due to the stringent parameters that we used to ensure a robust association between a truncated copy and a dated copy harboring two LTRs (see Materials and Methods), the insertion date could not be estimated for all truncated copies (Table 3).

Finally, our method allows an extensive characterization of LTR retrotransposon copies in today's rice genome, as well as their temporal dynamics of insertion and deletion, leading to a comprehensive analysis of the processes involved in their evolutionary dynamics in the rice genome.

Our results are based on the assumption that the copies extracted are derived from the insertion of complete copies. This assumes that the mechanism of retrotransposition do not lead to truncated copies upon integration. This mechanism has been well studied in retroviruses, and it is commonly accepted that it also applies to LTR retrotransposons. To our knowledge, insertion of truncated copies has not been described, meaning that if this is possible, it would occur rarely. Consequently, we believe that the frequency of such events is low, and should not have a large impact on our results.

During our analysis, we revealed for *Hopi/Osr27 *and *Osr8 *the presence of truncated copies sharing a deleted region compared to the reference copy. These two subsets of copies also share similar insertion times, suggesting that they correspond to the amplification of truncated copies. Since this was observed for 2 elements only, we believe that this process does not affect tremendously our conclusions on the overall deletion process. Moreover, since the formation of small deletions is a cellular process and should therefore affect similarly all types of elements, the conclusions drawn on the deletion rate are not affected by this observation.

## Conclusion

Through the analysis of 41 rice LTR retrotransposon families, and the use of a new method to estimate the insertion date of both truncated and complete copies, we could precisely describe the amplification and elimination of LTR retrotransposon sequences in the rice genome (*O. sativa *L.). We show that most of the copies were inserted within the last 2 My, and that the amplification process varies both in timing and extent between families. The copies are subsequently efficiently eliminated from the genome, through both solo LTR formation and accumulation of deletions. We estimate the half-life of LTR retrotransposon sequences in the rice genome to be less than 3 My. However, if only the neutral accumulation of small deletions is taken into account, this half-life would be close to 19 My, revealing that this process is not a major force of LTR retrotransposon removal in the rice genome. Rather, negative selection of these sequences or larger rearrangements may be involved. Altogether, these results reveal a high turn-over of LTR retrotransposon sequences in the rice genome and therefore provide an explanation for the rapid differentiation of intergenic regions in grass genomes. To our knowledge, our study is the first to analyze rice LTR retrotransposons in such an extensive way.

## Methods

### Mining of LTR retrotransposons in the rice genome

We started by compiling a dataset containing all the rice LTR retrotransposon sequences available from the literature [28,44,45] and from the public databanks [46,47]. We subsequently updated this dataset with the LTR retrotransposon sequences that we had previously characterized [29,48]. We then eliminated the redundancy of this first dataset by performing dot plot comparisons, leading to a final non redundant database containing 47 LTR retrotransposon sequences. Through this search, we found that *Osr13 *is *Houba/Osr13*, *Osr25 *is *Dasheng*, *Osr26 *is *Retrosat1/RIRE2*, *Osr27 *is *Hopi/Osr27*, *Osr33 *is *RIRE8*. Some of these annotations were in agreement with ref 28, Table 1, some were not. Moreover, we could not find any significant similarity between *RIRE1 *(from Genbank accession D85597) and *Osr11*, between *Osr27 *and *RIRE9 *(from Genbank AB033547) or between *Tos17 *and *Osr21*, as stated by other authors (28). Since this paper did not clearly states how the "pre-existing names" were associated with the "Osr" elements, we decided to follow our annotation, not the one published (28), and kept the Osr nomenclature for the elements that we did not annotate. We then carefully analyzed the boundaries of each retrotransposon, and corrected then when needed. For each LTR retrotransposon reference copy where LTRs were detected, the two LTRs were homogenized by replacing the 3' LTR by the sequence of the 5' LTR, to avoid differences in match between the two LTRs. A fasta file of the sequences of the 41 LTR retrotransposon reference copies contained in this database is available in additional file 1 on the BMC Genomics website.

To annotate possible insertions in the LTR retrotransposon genomic sequences that we would retrieve, we completed this dataset with known rice TEs such as DNA transposons, Miniature Inverted-repeat Transposable Element (MITE) sequences and partial LTR retrotransposon sequences that were available in the public databanks [46,47]. Similarly, three LArge Retrotransposon Derivatives (LARDs) (*Osr25/Dasheng*, *Spip *and *Squiq*) and their autonomous counterparts (*Retrosat1/RIRE2/Osr26 *[49], *RIRE3 *and *RIRE8/Osr33*, respectively [48]) contained in our dataset were used only for the annotation of genomic copies. Indeed, the high sequence identity shared between the LTR sequences of each LARD/retrotransposon couple could have induced ambiguous annotation of the genomic copies, and notably artefactually increase the number of LTRs detected for these two families. Hence, the final dataset that we further analyzed contained 41 LTR retrotransposons, including 16 *Gypsy*-like and 25 *Copia*-like families that varied in element and LTR size (Table 1).

We extracted the copies of these 41 LTR retrotransposons in the rice genome sequence using Blaster, a program suite based on the BLAST program [35] and improved for the detection of transposable elements [4]. For the current study, we first performed a nucleotide-nucleotide BLASTN search, using the 12 rice (*Oryza sativa *cv. Nipponbare) pseudomolecules (representing almost 365 Mb, (*i.e *~93% of the entire genome) as query and our transposable elements dataset (containing LTR retrotransposons, LARDs complete sequences, LTR retrotransposons partial sequences and MITEs sequences, see above) as subject. We then filtered the resulting High Scoring Pairs (HSPs), using a 1 × 10^-10 ^E-value threshold. Finally, the selected HSPs were connected by means of a dynamic programming algorithm based on HSP scores and the physical distance that separated them (Blaster suite, [4]). Thus, pieces of retrotransposons that are separated by large insertions (such as insertions of other TEs) can be connected together in the genomic sequence. Extracted copies whose sequence harbored stretches of N, indicating the presence of unfinished sequences, were then eliminated in order not to bias the estimation of copy size and the classification of the copies (see below for detail of these procedures).

### Characterization of the LTR position for the 41 LTR retrotransposons

The precise localization of the LTR was a key step in our study, both for the classification of the copies based on their truncation level and the estimation of their insertion date. Therefore, we carefully checked the position of the LTRs of the 41 non redundant reference copies. This was performed as follows: we first extracted the data concerning LTR position in the literature. We then annotated the LTR position by (1) aligning each reference sequence with itself, using a "BLASTN 2 sequences" analysis [50] and (2) comparing the first half of the sequence with the second half, using Smith and Waterman local alignment, thus making it possible to detect repeated regions of the same orientation within each reference copy. We then compared the results (data not shown). When at least two methods out of the three gave the same results, we considered the position to be accurate and kept the element for further analysis (names in bold face in Table 1). Hence, we did not analyze 7 elements any further, either due to a lack of any observable LTRs whatever the method (*Osr9*, *Osr10, Osr11*, *Osr18*) or because our analysis did not confirm the LTR positions described in the literature (*Osr16*, *Osr23*, *Osr31*). In Table 1, these elements are referred to as "RT only" and "ambiguous LTR position".

### Analysis of short copies and size filter

Because the reference copies are derived from genomic copies, we expected some of them to contain uncharacterized insertions, such as unknown MITEs or pieces of unknown transposable elements. For this reason, certain short copies retrieved by our Blaster search could correspond to paralogous copies of these TEs, and not to the LTR retrotransposons of interest. To check for such possible insertions in the reference copies, we aligned all the mined genomic copies on their corresponding reference copies, and looked for subsets of copies that were a complete match with the same region of the reference copy. This allowed us to spot sub-regions of the reference copies that were highly repeated thus revealing inaccurate boundaries for *Osr4*, presence of a LARD element related to *Osr14*, as well as insertions in some reference copies. Two types of inserts were found: inserts with a size over 200 bp in reference copies *Osr1*, *Osr5*, *Osr22*, *Osr34*, *Osr37*, *Osr39*, *Osr40 *and smaller inserts in many other copies.

To discard these false-positive genomic copies that would lead to an over-estimate of the deletion rate, we filtered out every fragment with a size below 200 bp, and did not further analyze the 9 families for which insertions larger than 200 bp, or a related LARD element, were found. Families which LTR size is shorter or close to 200 bp (*Osr19*, *Osr44*, *Tos17*, *Osr20*, *Osr24*, *Osr43*) were also discarded, because the filter could lead to an under-estimation of LTR copy number. Finally, we thus conducted a precise analysis of the deletion process for 19 families (10 of *copia *type and 9 of *gypsy *type).

### Classification of the copies according to their truncation level

For each genomic copy, Blaster produces an alignment (connected HSPs) showing the coordinate correspondence between each genomic copy and the reference sequence. Therefore, if the position of the LTR is annotated in the reference copy, the position of the LTR (if it exists) can be determined for each genomic fragment. Using the LTR position of our reference sequences, and the alignments generated for each genomic copy by Blaster, we thus classified the genomic copies as "2 LTRs", "LTR-IR", "LTR", and "IR" (Internal region). Note that the presence of a LTR or an internal region does not necessarily mean that it is complete, since it can harbor internal deletions.

### Copy size

To estimate the global genomic part corresponding to the 41 families, we extracted the length of each copy as given by the Blaster search. For subsequent analysis, we needed to estimate the copy length more accurately and thus did not want to take into account small stretches of mismatch that could have been excluded in the Blaster length computation. For our detailed analysis of the deletion process, we therefore computed the copy length by extracting the sequence of each copy, the sequence from Blaster start position to Blaster end position, and then aligning each copy with its corresponding reference copy, using the gap global alignment program [51], with the following parameters: matches (+10), mismatches (-7), gap open (-16), gap extension (-6). We chose these parameters to allow for long gaps (possibly corresponding to large insertions/deletions) in the alignments. The length was then computed by counting the positions of all matches and mismatches in the alignment.

### Insertion date

We estimated the insertion date of the complete copies using the method proposed by SanMiguel and co-workers [9], which is based on the divergence between the two LTRs of the copy. Due to the LTR retrotransposon replication cycle, when a new copy inserts in the genome, its two LTRs are identical in sequence. As time elapses, the two LTR sequences accumulate mutations and thus diverge from each other. This divergence can be converted into an insertion date by the use of a substitution rate, with the equation: T = D/2t, where T is the time elapsed since the insertion, D the estimated LTR divergence and t the substitution rate per site per year. The observed divergence between LTRs was corrected for homoplasy using the Jukes and Cantor model [52] and the substitution rate that we used was 1.3 10^-8 ^substitutions per site per year, as proposed by Ma and Bennetzen [53] after calibrating the substitution rate of the *adh *genes [54] to rice LTR retrotransposons. This divergence was calculated only for copies where at least 100 bp could be aligned between the two LTRs. For the copies lacking at least one LTR, we could not use this method. Therefore, we estimated their insertion date as follows: we used all the truncated copies as query against all the complete copies using a Blaster search. Each truncated copy was therefore coupled to the complete copy with the best match, and the insertion date of this complete copy was used to estimate the insertion date of the truncated one. When the best score was shared by more than one complete copy, the date of the truncated copy was estimated only if the LTR divergence (*i.e*., the age) of the oldest and youngest of these matching complete copies did not differ of more than 1%, to ascertain that these complete copies originated from the same amplification burst. This procedure allowed us to purge any fragments that originated from recombination events involving two copies with different insertion times. In this case, the age taken into account was the one of the youngest copy.

The insertion date of the truncated copies was computed as follows: the nucleotide divergence between the truncated copy and the best matching complete copy was computed, and compared to the nucleotide divergence existing between the two LTRs of the complete copy. If these two copies originated from the same amplification burst, these two numbers should be similar (Figure 1). They were considered similar if they did not differ of more than 1%.

### Deletion rate estimates

To estimate the deletion rate, we used the maximum likelihood approach proposed by Petrov *et al. *[41,42]. This method is based on the relative ratio of deletions versus nucleotide substitutions. According to the assumptions that (1) the rates of deletion and substitution do not vary over time and (2) at any given time, the number of substitutions and deletions follows a Poisson distribution, a maximum likelihood estimator can be calculated. The confidence limits can be found using the χ^2 ^approximation of log-likelihood ratio. A modified version of this estimator, that corrects for the sizes of the deletions and the sizes of the sequences in which they occurred, has subsequently been proposed [43]. To compare the two methods, we estimated the deletion rate given by these two estimators on several samples from different species including rice (data not shown). Both lead to nearly identical results, indicating that deletion size are small compared to the sequences in which they occur. Considering this, we performed our final analysis using the uncorrected estimator, because its confidence limits can be easily computed.

Multiple alignments for each family were obtained by first computing the pairwise global alignment with the reference sequence using the gap program [51], and then stacking all pairwise alignments to obtain a master-slave multiple alignment. Regions that were not present in the reference sequence were removed from the multiple alignments, so insertions were not taken into accounts. Finally, sequences that could not be properly aligned with the rest of the set were removed. Trees were built using the PHYLIP 3.5c package [55]. For each alignment, we built a distance matrix correcting for multiple substitutions with the Kimura two-parameters model [56]. Neighbor-joining algorithm was used to build the phylogeny.

From these phylogenies, we extracted only the terminal forks and not all the terminal branches as in [41,42]. This improves the methods at several key points: first, any gap present in the alignment of the two sequences of a fork has appeared after the divergence of these two sequences. Therefore, deletions are unambiguously assigned to a time scale, estimated by the branch length. Second, the size of the sequence where the deletion occurred corresponds to the size of the pairwise alignment between the two sequence pairs (excluding parts of the alignment corresponding to terminal gaps), and can therefore be accurately monitored. Third, the divergence time between these paired sequences being shorter than between sequences from different terminal branches, deletion estimates are less biased by possible gaps aggregations (over long time periods, the observation of a large gap could be the result of one large deletion, or several recurrent deletions of smaller extent).

The a half-life of a sequence was calculated using a continuous decay formula, as in [42]: *L *= *L*_0_*exp(-rt)*, where *L *is the length of the sequence at time *t*, *L*_0 _is the length at time 0, and r the product of the average size of a deletion by the rate of deletions per substitution or per year.

## Abbreviations

HSP: High Scoring Pair

IR: Internal Region

TE: Transposable element

LARD: LArge Retrotransposon Derivative

LTR: Long Terminal Repeat

MITE: Miniature Inverted Transposable Element

RDA: Representational Difference Analysis

## Authors' contributions

CV participated in the design of the study, carried out the mining of the genome and the characterization of the LTR retrotransposon copies (size, degree of truncation, insertion date), analyzed the corresponding data, and drafted the manuscript. OP participated in the design of the study, in the analysis of the data and helped to draft the manuscript. HQ participated in the design of the study, performed the phylogenetics and statistical analyses for the deletion rate estimate, analyzed the data and helped to draft the manuscript.

## Supplementary Material

Additional file 1 **Sequences of the 41 LTR retrotransposon reference copies**. Sequences can be uploaded in fasta format from the "Vitte_et_al_reference_copies.txt" text file. Note that reference copies are derived from genomic copies. Their LTR sequences have been homogenized by replacing the two LTR sequences by the sequence of the 5' LTR, to avoid detection problems, except for *Osr9*, *Osr10*, *Osr11*, *Osr16*, *Osr18*, *Osr23*, and *Osr31 *for which the position of the LTR was ambiguous (see text and Table 1 for details). Click here for file
